# A Convenient and Effective Method to Deposit Low-Defect-Density nc-Si:H Thin Film by PECVD

**DOI:** 10.1186/s11671-018-2641-z

**Published:** 2018-08-10

**Authors:** Yuwei Wang, Hong Liu, Wenzhong Shen

**Affiliations:** Key Laboratory of Artificial Structures and Quantum Control (Ministry of Education), Department of Physics and Astronomy, Shanghai Jiao Tong University, Shanghai, 200240 People’s Republic of China

**Keywords:** Hydrogenated nanocrystalline silicon, PECVD, Deposition pressure, Ion bombardment, Defect density

## Abstract

Hydrogenated nanocrystalline silicon (nc-Si:H) thin film has received a great deal of attention as a promising material for flat panel display transistors, solar cells, etc. However, the multiphase structure of nc-Si:H leads to many defects. One of the major challenges is how to reduce the defects conveniently. In this work, we developed a simple and effective method to deposit low-defect-density nc-Si:H thin film. This method is simply by tuning the deposition pressure in a high-pressure range in plasma-enhanced chemical vapor deposition (PECVD) process. Microstructures of the nc-Si:H were characterized by Raman, AFM, and SEM. Furthermore, we focused on the defect density which was the key characteristic for photovoltaic materials and achieved the defect density of 3.766 × 10^16^ cm^−3^. This defect density is lower than that of previous studies on the fabrication of low-defect-density nc-Si:H by other complex methods in PECVD process. The minority carrier lifetime of nc-Si:H is thus greatly improved. Moreover, we demonstrated the mechanism about the effect of deposition pressure on the ion bombardment and proved that the defect density is the key characteristic for nc-Si:H photovoltaic material.

**Electronic supplementary material:**

The online version of this article (10.1186/s11671-018-2641-z) contains supplementary material, which is available to authorized users.

## Background

An important landmark in the progress of thin film silicon technology is the development of high-quality hydrogenated nanocrystalline silicon (nc-Si:H). Compared with hydrogenated amorphous silicon (a-Si:H), nc-Si:H has much higher mobility, much better response at wavelengths greater than 800 nm, and is much less susceptible to the Staebler-Wronski degradation [1, 2]. nc-Si:H thin film can be deposited using plasma-enhanced chemical vapor deposition (PECVD), which makes it compatible with well-developed integrated circuit industry. Therefore, nc-Si:H thin film has a wide application in various devices, such as thin film transistors [3], photodetectors [4], and solar cells [5].

However, as a multiphase material, nc-Si:H thin film has its own defects such as voids and dangling bonds at the interfaces of crystals/amorphous phase and between crystals. It is known that the atomic hydrogen is the key to the deposition of high-quality nc-Si:H which has less defects [6]. The atomic hydrogen can saturate dangling bonds, and it has been pointed out [7] that the atomic hydrogen on the growing surface gives rise to crystal growth at a temperature much lower than the melting one. Thus, more atomic hydrogen is beneficial for the deposition of high-quality nc-Si:H. In order to increase the atomic hydrogen flux on the growing surface, high hydrogen dilution or silane depletion would be essential for nc-Si:H growth.

Yielding silane depletion in PECVD is to raise radio frequency (RF) power [7]. But simply increasing the power will dramatically increase the ion bombardment on the growing surface, which will probably lead to more defects. Thus, a direct current (DC) bias should be used to suppress the ion bombardment. However, if the RF power is increased to obtain more atomic hydrogen, the DC bias should be changed as well. Otherwise, the DC bias cannot relieve the ion bombardment effectively. And the suitable DC bias under certain RF power cannot be found without the vast experiments. Another method of raising the atomic hydrogen content is to increase the deposition pressure. Electron-molecule collision frequency increases with the deposition pressure. It makes the dissociation rate of SiH_4_ and H_2_ rising. A dissociation of SiH_4_ and H_2_ generates the atomic hydrogen [8]. As a result, increasing deposition pressure could raise the atomic hydrogen content.

In this paper, we thus propose to tune the deposition pressure in a high-pressure range to obtain more atomic hydrogen (the conventional deposition pressure is 50–100 Pa). It is a convenient and effective method in the PECVD process. By using this method, the film deposited under a certain pressure has a lower defect density in comparison with previous studies on the fabrication of low-defect-density nc-Si:H [5, 9, 10]. And a high minority carrier lifetime has been achieved. In addition, compared with the previous reports on the effect of different deposition pressures on the macroscopic or general properties of the samples [11, 12], we have significantly extended the range of deposition pressure and focused on its effect not only on the general properties (e.g., crystallinity) but also on the defect density and minority carrier lifetime which are key characteristics for high-quality photovoltaic material. Furthermore, we have demonstrated the mechanism about the effect of deposition pressure on the ion bombardment, while previous reports just gave general discussions. And we have further proven that the ion bombardment is not the weaker the better for the film growth (the degree of ion bombardment should be appropriate). In the end, we have proved that the defect density is the key characteristic for nc-Si:H photovoltaic material.

## Methods/Experimental

**Fig. 1:**
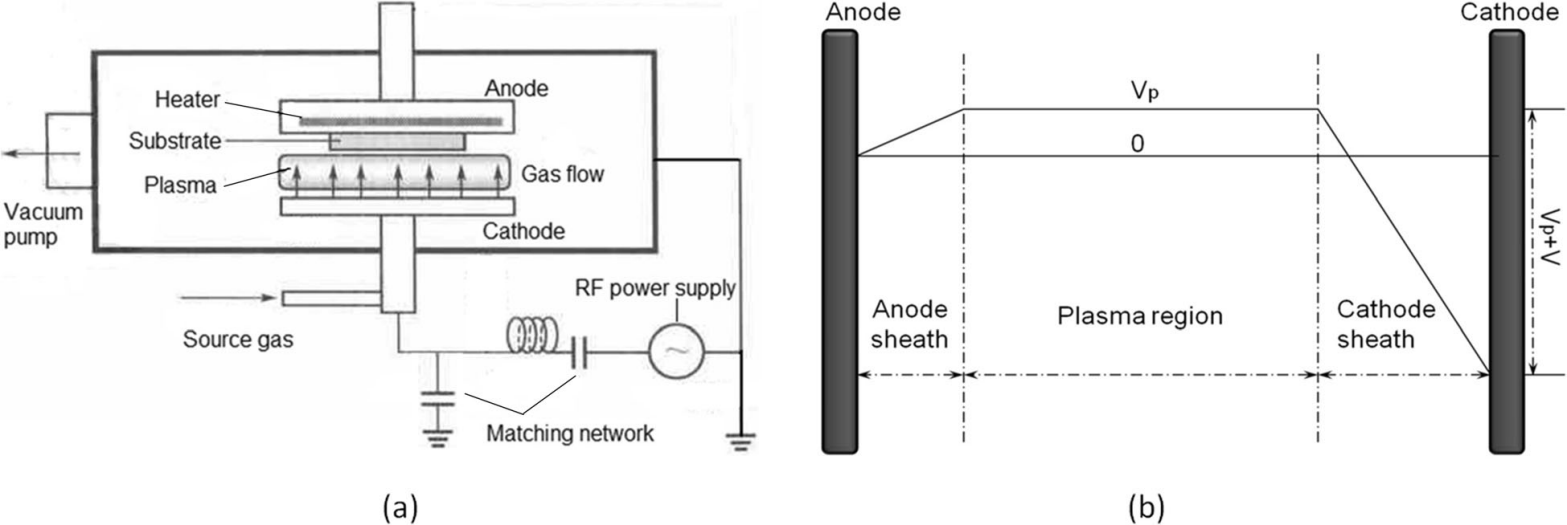
The schematic diagram of **a** the PECVD reactor and **b** the distribution of potential between electrodes (*V*_*p*_, the plasma potential; *V*, the root mean square RF potential)

The nc-Si:H thin films were grown on Corning glass by a capacitively coupled PECVD system (the schematic diagram of the reactor is shown in Fig. 1a) at different deposition pressure. The deposition pressure was increased from 150 to 1050 Pa, with a step of 150 Pa. All the samples were deposited using RF of 13.56 MHz and power density of 0.32 W/cm^2^, with a total gas (SiH_4_ and H_2_) flow rate of 110 sccm (the SiH_4_ concentration was 0.727%). The substrate temperature was kept at 250 °C, and the deposition time was 2 h.

The crystallinity *X*_*c*_ was calculated from Raman spectra measured with a UV micro-Raman spectrometer (Jobin Yvon LabRam, HR800) in backscattering mode using an Ar-ion laser at 514.5 nm. The laser power density was kept at 1 mW/mm^2^ to avoid any beam-induced crystallization. The defect density of the samples was characterized by the spin density Ns, which was calculated from the results measured by an electron spin resonance (ESR) spectrometer (Bruker, EMX-8X-band) at 9.8 GHz and 5 mW. The effective minority carrier lifetime *τ* was measured using a Semilab WT-1200A. The surface morphology of these films was observed by an atomic force microscope (AFM, SII Nanonavi E-Sweep), and the micromorphology was observed by a scanning electron microscope (SEM, Sirion 200).

## Results and Discussion

### Structural Investigation by Raman Analysis

**Fig. 2:**
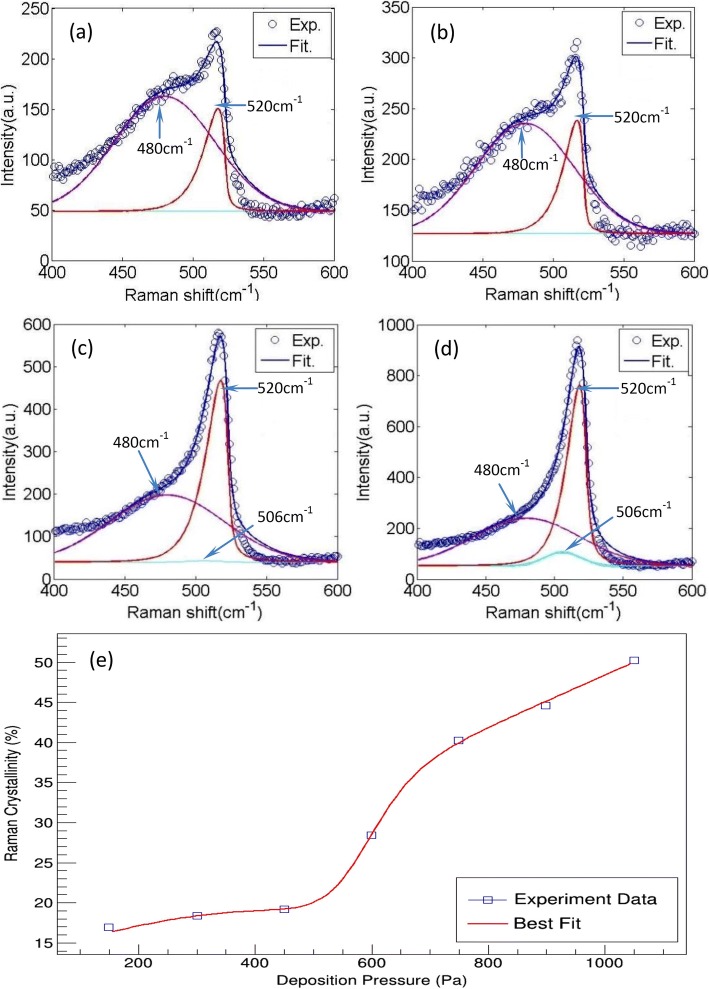
Raman spectra and their deconvolution of typical samples deposited under 300 Pa (**a**), 450 Pa (**b**), 750 Pa (**c**), and 1050 Pa (**d**) and the calculated crystallinity of nc-Si:H thin films deposited under different pressures (**e**)

For the structural investigation of the nc-Si:H thin film deposited under various pressure, micro-Raman measurements were carried out. In Fig. 2, four representative samples were chosen to show the Raman spectra. They are deposited under 300 Pa, 450 Pa, 750 Pa, and 1050 Pa, respectively. Each spectrum (open circles) under certain pressure can be deconvoluted into three Gaussian peaks: (1) a broad Gaussian distribution around 480 cm^−1^, which is attributed to the transverse optical (TO_1_) mode of amorphous silicon; (2) a peak near 520 cm^−1^, which belongs to the asymmetric TO_2_ vibrational mode of crystalline silicon [13, 14]; and (3) the peak around 506 cm^−1^ which is attributed to the intermediate range order [1, 15]. The crystallinity (*X*_*c*_) in nc-Si:H can be calculated by [16, 17]:

1 $$ {X}_c=\left({I}_{520}+{I}_{506}\right)/\left({I}_{520}+{I}_{506}+\gamma {I}_{480}\right) $$

where *γ* is the ratio of the integrated Raman cross-section for c-Si to a-Si (*γ* = 1 [17, 18]), and *I*_520_, *I*_506_, *I*_480_ are the integrated intensity of the peaks observed at 520, 506, 480 cm^−1^, respectively. The crystallinity as a function of different deposition pressures is plotted in Fig. 2e.

It is known that the atomic hydrogen (H), is mainly generated from the following two collisions in hydrogen diluted silane plasma [8]:

- The primary electron-silane reaction

2 $$ {\mathrm{SiH}}_4+\mathrm{e}\to {\mathrm{SiH}}_3+\mathrm{H} $$

b. The electron-hydrogen reaction

3 $$ {\mathrm{H}}_2+\mathrm{e}\to \mathrm{H}+\mathrm{H} $$

According to formulae S(1) and S(2) in the Additional file 1, we can get ${n}_e=\frac{P}{RT}\bullet \mu$ (*P*, deposition pressure; *R*, ideal gas constant; *T*, absolute temperature of gas before discharge; *μ*, ionization rate; and *n*_*e*_, electron density). *μ* is constant because of the unchanged RF power, and *T* is constant as well. The electron density *n*_*e*_ thus increases with the deposition pressure *P*. According to chemical Eqs. (2) and (3), the density of H which is produced in the plasma increases with *n*_*e*_. This is the theoretical analysis in an ideal condition. Discharge process is so complex that the analysis on discharge process (i.e., plasma diagnoses) becomes an independent discipline. The change of H with the deposition pressure in the real condition should be measured through plasma diagnoses. Yang et al. measured the intensity of ${H}_{\alpha}^{\ast }$ (${I}_{H_{\alpha}^{\ast }}$) by optical emission spectra (OES) and demonstrate ${I}_{H_{\alpha}^{\ast }}$ first increases then decreases [19]. According to previous plasma diagnoses reports, the intensity of ${H}_{\alpha}^{\ast }$ indicates the amount of atomic hydrogen [20, 21]. Thus, the density of H in the plasma first increases then decreases when the deposition pressure continues increasing. This trend is a little different to our theoretical analysis. The difference is related to the secondary reaction of H:

4 $$ \mathrm{H}+{\mathrm{SiH}}_4\to {\mathrm{H}}_2+{\mathrm{SiH}}_3 $$

where SiH_4_ is the one which is not decomposed, i.e., the remaining SiH_4_. In our experiment, the deposition pressure is increased by reducing the outflow of gases including SiH_4_. In other words, it is equivalent to increase the supplement of SiH_4_.When the deposition pressure rises to a certain level, the speed of SiH_4_ supplement exceeds its decomposition speed. Thus, there are more amounts of SiH_4_ left. There is a distance for H from its escape out of the plasma to its arrival at the film-growing surface. H will react with the remaining SiH_4_ in this distance, as shown in the secondary chemical Eq. (4). The density of H thus decreases. As a result, the atomic hydrogen density first increases then decreases when the deposition pressure continues increasing. It is known that more amount of H is beneficial to deposition of low-defect-density nc-Si:H. Therefore, the defect density of nc-Si:H deposited in our experiment shows the same tendency of the atomic hydrogen density, i.e., the defect density first decreases then increases. The discussions about the trend of defect density in detail are shown in the latter section.

In Fig. 2e, it can be clearly seen that the crystallinity of nc-Si:H, *X*_*c*_, increases with the deposition pressure. This indicates that increasing pressure can raise *X*_*c*_. The crystallinity is not only affected by the atomic hydrogen but also influenced by the content of growth precursor SiH_*n*_ (*n* = 1,2,3, mainly *n* = 3) which can be indicated by SiH^*^ in OES measurement [21, 22]. Hsieh et al. have demonstrated that ${I}_{H_{\alpha}^{\ast }}$/*I*_SiH_^∗^ (the intensity ratio ${H}_{\alpha}^{\ast }$/SiH^*^) increases with the deposition pressure [20]. It is generally accepted that ${I}_{H_{\alpha}^{\ast }}$/*I*_SiH_^∗^ is the index for *X*_*c*_, i.e., *X*_*c*_ increases with the increase of ${I}_{H_{\alpha}^{\ast }}$/*I*_SiH_^∗^ [21, 23]. Therefore, the ${I}_{H_{\alpha}^{\ast }}$/*I*_SiH_^∗^ trend strongly support our result about the tendency of *X*_*c*_.

The mean grain size *d* can also be deduced from Raman spectrum, according to the formula [24, 25]:

5 $$ d=2\pi \sqrt{B/\Delta  \upnu} $$

where *∆ν* is the frequency in unit of cm^−1^ shift, which was defined as the difference between the observed peak frequency value and that of the bulk Si. Using the usual value of *B* of 2.0 cm^−1^ nm^2^ [25], *d* = 4.07~4.50 nm.

### Surface Morphology and the Mechanism About the Influence of the Deposition Pressure on the Ion Bombardment

**Fig. 3:**
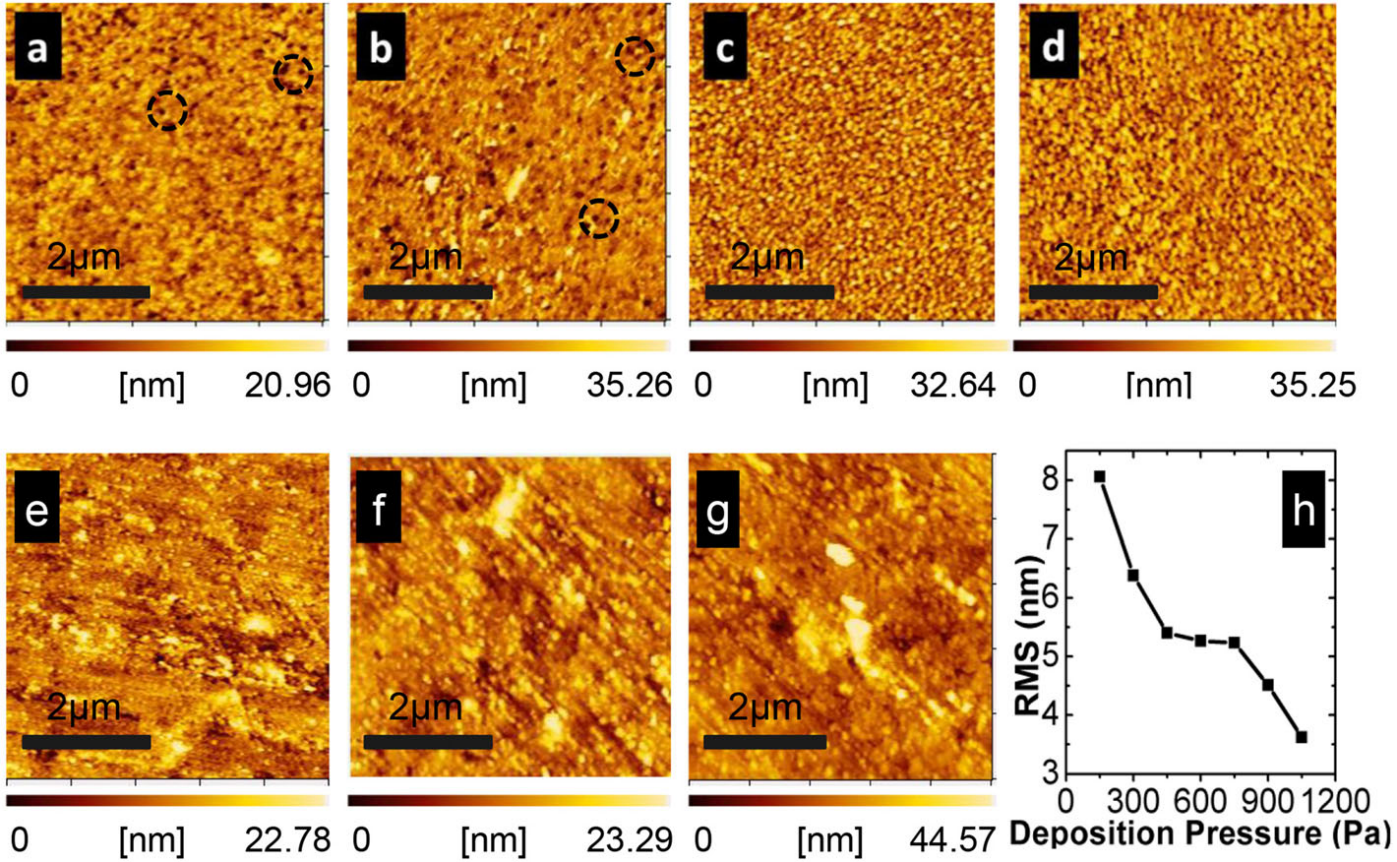
AFM images of nc-Si:H thin films showing a surface morphology change by different deposition pressures. **a** 150 Pa, **b** 300 Pa, **c** 450 Pa, **d** 600 Pa, **e** 750 Pa, **f** 900 Pa, and **g** 1050 Pa. The craters in **a** and **b** are marked by dashed circles, and root mean square (RMS) roughness of film surfaces under different deposition pressures marked as **h**

Besides the structural analysis by Raman spectroscopy, the morphology of the samples was also characterized by AFM, as shown in Fig. 3. To detect the roughness evolution of the film surfaces, the root mean square (RMS) as a function of deposition pressure was depicted in Fig. 3h (RMS value was averaged over several different locations in each film). In Fig. 3h, RMS decreases as the deposition pressure increases. The increasing pressure causes an aggravated collision between particles and subsequent loss of kinetic energy when these particles reach the film-growing surface. Lower energy ions arriving at the film-growing surface lead to weaker ion bombardment. It suggests that the increasing pressure is beneficial to suppression of the ion bombardment, which has been also mentioned in the previous report [7]. However, the mechanism about the influence of the deposition pressure on the ion bombardment has not been demonstrated. It will be investigated as follows.

The potential distribution between the two electrodes can be divided into three regions: plasma region in the center, anode sheath, and cathode sheath (see Fig. 1b). The ions which lead to ion bombardment must diffuse out of the plasma region and pass through the anode sheath. The potential of plasma is higher than that of any other sections of the reactor because electrons diffuse faster than ions. As our reactor wall is grounded, the potential of plasma is positive (see Fig. 1). Anions are thus trapped in the plasma region; only the neutral particles and cations can diffuse to the anode sheath and finally reach the film-growing surface. In other words, the ion bombardment in our experiment is caused by cations only. Cations pass through the anode sheath without collision as the width of the sheath is very small (the evidence in detail is shown in Additional file 1). As a result, cations are only accelerated by the electric field of the anode sheath when they enter the sheath. Therefore, the strength of ion bombardment depends only on cation’s initial velocity when they just enter the anode sheath (*v*_0_) and the degree of acceleration by the electric field of anode sheath afterward.

Firstly, the correlation of *v*_0_ with the deposition pressure will be analyzed. Particles including cations lose their kinetic energy in the plasma region due to the aggravating collision when the deposition pressure increases. So, *v*_0_ declines as the pressure increases. Then, the variation in the degree of acceleration by the electric field of the sheath with the deposition pressure will be demonstrated. It is known that [22]:

$$ {V}_p-{V}_f=\frac{k{T}_e}{2e}\left(\frac{m_i{T}_e}{m_e{T}_i}\right) $$

where *m*_*e*_ is the electron’s mass; *m*_*i*_ is ion’s mass; *T*_*e*_ and *T*_*i*_ are the temperature of electron and ion, respectively; *V*_*p*_ is the plasma potential; and *V*_*f*_ is the floating potential. As the substrate is suspended in our reactor, the voltage of the anode sheath, *V*_sheath_ is equal to *V*_*p*_ − *V*_*f*_, then we have:

6 $$ {V}_{\mathrm{sheath}}=\frac{k{T}_e}{2e}\left(\frac{m_i{T}_e}{m_e{T}_i}\right) $$

**Fig. 4:**
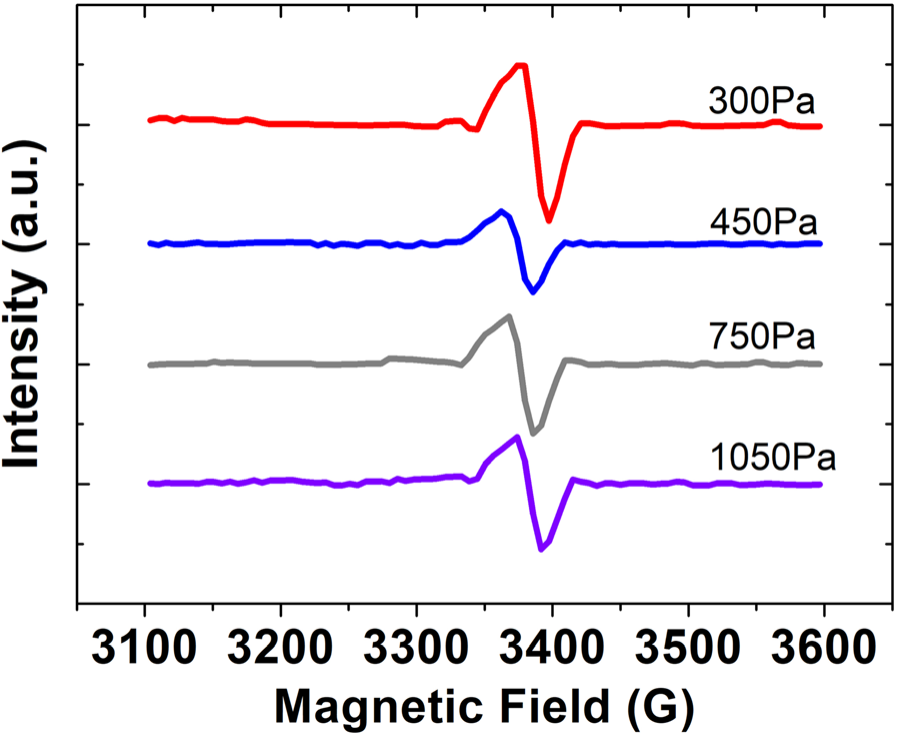
The ESR spectra of typical samples deposited under 300 Pa, 450 Pa, 750 Pa, 1050 Pa

**Fig. 5:**
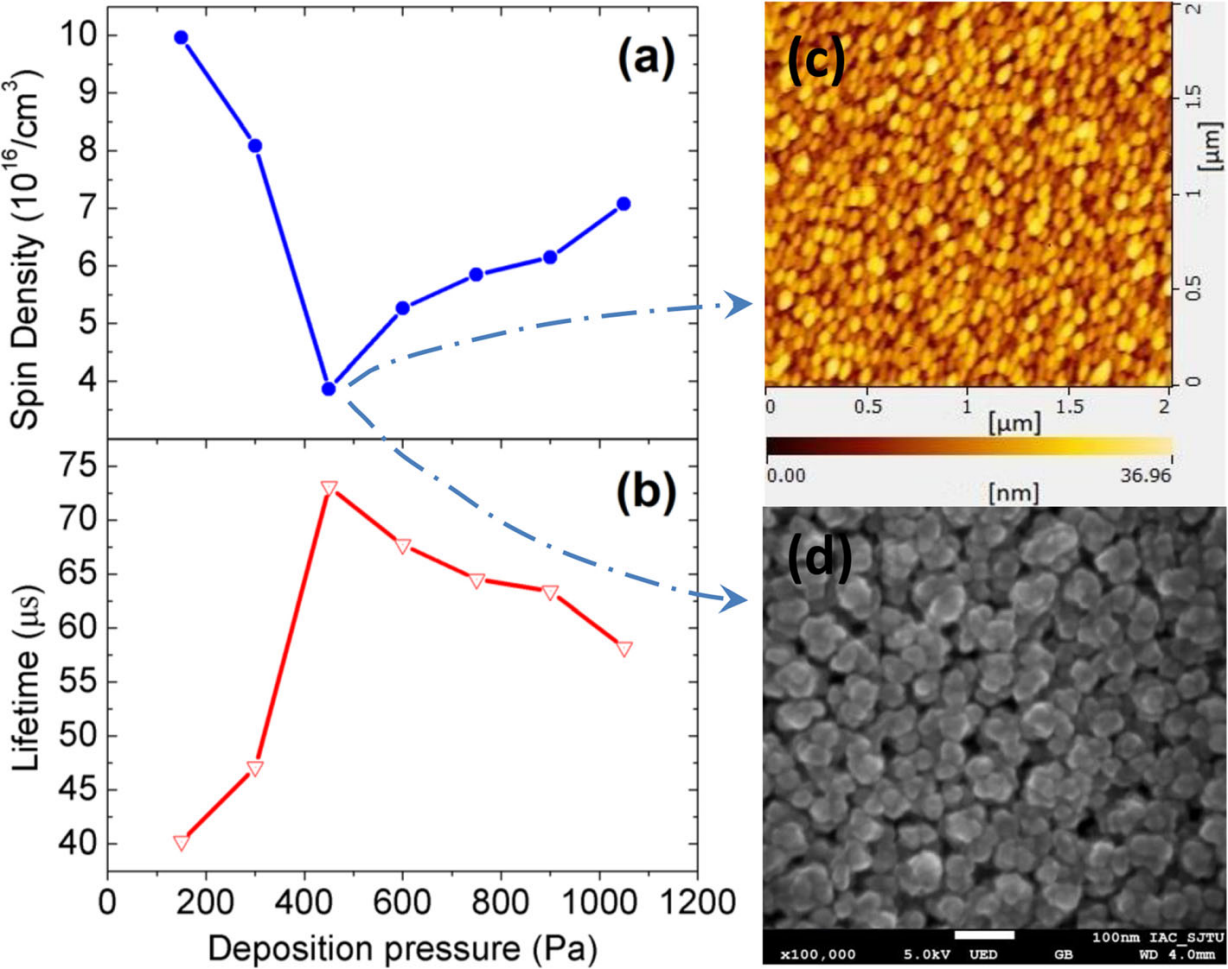
Dependence of spin density (**a**) and effective minority carrier lifetime (**b**) on different deposition pressures. The high-resolution AFM image (**c**) and SEM image (**d**) of nc-Si:H thin film deposited under 450 Pa

**Fig. 6:**
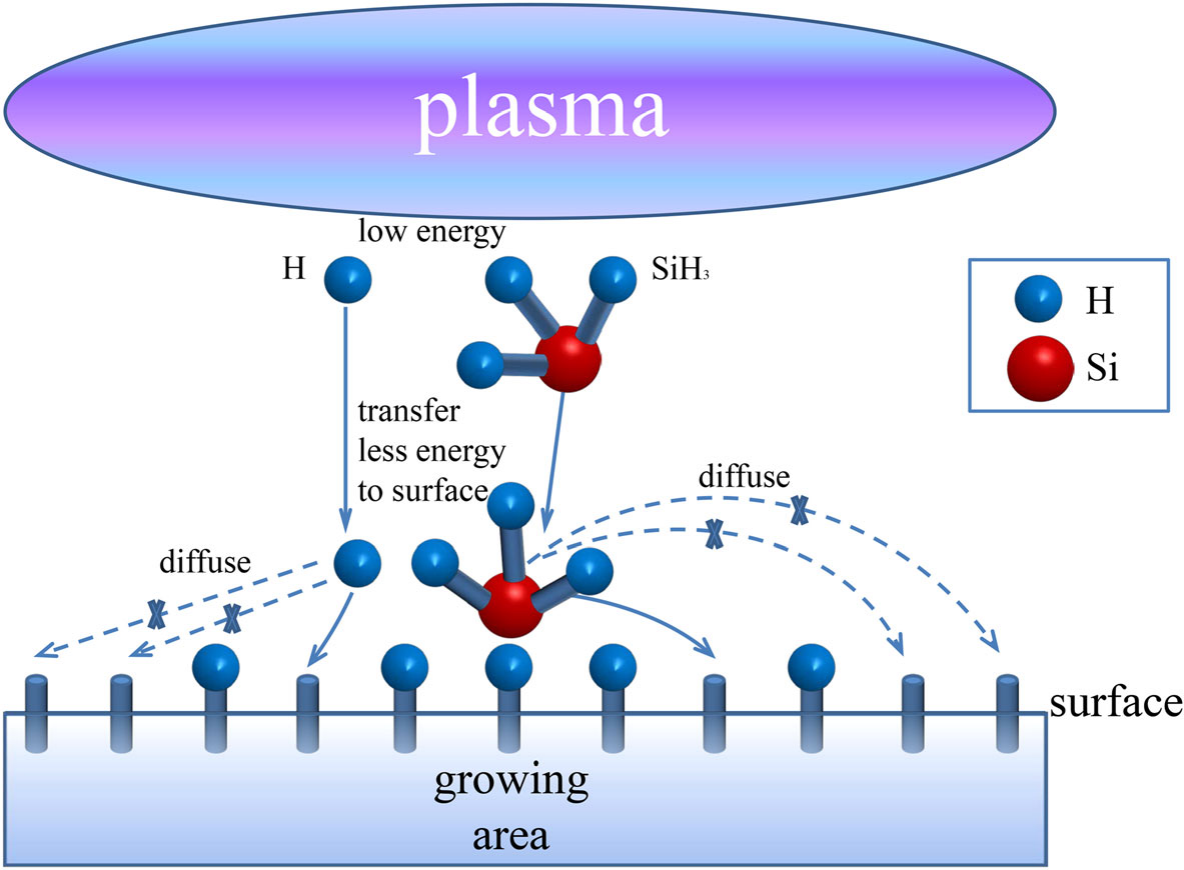
Schematic diagram of particle diffusion on the growing surface

In the plasma region, *T*_*e*_ decreases as increasing deposition pressure aggravates the collision between the particles (including electrons and ions). Hsieh et al. have demonstrated that *T*_*e*_ decreases with the increasing deposition pressure by the OES measurement [20]. This suggests that the trend of *T*_*e*_ by our theoretical analysis is absolutely correct. Compared with *T*_*e*_, *T*_*i*_ decreases so little that it can be considered unchanged. As a result, *V*_sheath_ declines when the pressure increases according to Formula (6). It weakens the degree of acceleration by the anode sheath. Coupled with falling *v*_0_, we can draw a conclusion that the kinetic energy of cations that reaches the film-growing surface becomes much smaller when the pressure increases. In other words, increasing deposition pressure makes the ion bombardment effect weaker. Therefore, RMS of the film surface keeps decreasing from 150 to 1050 Pa. According to the previous report, the lower the ion energy, the better the crystallinity is [7]. It also supports the conclusion about the correlation between crystallinity and deposition pressure which we have already drawn. Moreover, the film surfaces which are deposited under much lower pressures (150 Pa and 300 Pa) are more rough, and we can see as well that these surfaces contain plenty of craters as shown in Fig. 4a, b. That is the consequence of strong ion bombardment. According to Fig. 3, we can apparently conclude that the film deposited under 450 Pa is the most compact (especially shown in Figs. 5c and 6d) and uniform.

### Defect Density and Electrical Property

The effect of the deposition pressure on the defect density of nc-Si:H thin film was investigated by ESR technique. Figure 4 shows the four ESR spectra of typical samples which were deposited under 300 Pa, 450 Pa, 750 Pa, 1050 Pa, respectively. The data of Fig. 5a are calculated from the ESR spectrum of each sample. As shown in Fig. 5a, when the pressure increases, the spin density first decreases then increases. There is a minimum at 450 Pa. According to the ESR principle, the number of unpaired spins is directly proportional to the density of neutral dangling bonds. These bonds mostly reside in the film-growing surface and constitute the steady-state defect of the film-growing surface, while the defect density in the resulting film is basically determined by these steady-state defect density [26]. Therefore, the results calculated from the ESR spectra are indeed the defect density of the resulting film. These results in Fig. 5a reveal that the defect density in nc-Si:H reaches a minimum at 450 Pa, which is 3.766 × 10^16^ cm^−3^. Chowdhury et al. studied how to fabricate low-defect-density nc-Si:H. When they used 13.56 MHz RF source, they did their best to achieve the low-defect density. The values were 1.1 × 10^17^ and 7.0 × 10^16^cm^−3^. When they used very-high-frequency (VHF) excitation source (54.24 MHz), they achieved the lowest defect density of 4.3 × 10^16^ cm^−3^ [10]. It is known that the ion energy in VHF plasma is low, and the density of ion flux is high. Due to these two factors, the thin film deposited by VHF-PECVD contains low-defect density and thus has high quality [27]. However, the defect density is higher than ours, and 54.24 MHz excitation source is much more expensive than its 13.56 MHz counterpart. In order to achieve low-defect density, Wen et al. additionally applied DC bias. However, the minimum is 4.0 × 10^16^ cm^−3^ [9]. Finding a suitable RF power is not easy, let alone an appropriate DC bias. The reason is that the DC bias should be tuned once the RF power is changed. Otherwise, the DC bias cannot relieve the ion bombardment effectively. By contrast, our method is simple. Recently, Jadhavar et al. have deposited a high-quality nc-Si:H by PECVD which has low-defect density. The defect density is about 8.75 × 10^16^ cm^−3^ [5]. Therefore, our method to achieve a low-defect-density thin film is convenient and effective. Taking it into consideration that the lower the defect density is, the higher the minority carrier lifetime should be, we directly carried out the measurement of the minority carrier lifetime. As shown in Fig. 5b, the effective minority carrier lifetime reaches its maximum at 450 Pa. The trend of minority carrier lifetime synchronizes with that of the defect density calculated from the ESR spectra. This reveals that the defect density of the films can be absolutely measured by ESR.

### Relationship of Ion Bombardment and Defect Density

Increasing pressure can suppress the ion bombardment. In a sense, the defect density should drift down continuously from 150 to 1050 Pa. In fact, it decreases then increases. There is another factor that should not be ignored—the diffusion of H and SiH_3_ (in the plasma, the main film precursor is SiH_3_ [26]). The schematic diagram of particle diffusion on the growing surface is shown in Fig. 6. From 150 to 1050 Pa, the kinetic energy of particles reduces. The ion bombardment effect is certainly lower and lower. However, the kinetic energy of particles reduces significantly from 450 to 1050 Pa due to the increasing particle collision frequency. The atomic hydrogen, which is the key to deposition of high-quality nc-Si:H, loses its kinetic energy so much that it cannot diffuse further to saturate more dangling bonds, let alone its density begins to decrease, which is shown in the “Structural investigation by Raman analysis.” On the other hand, particles, including atomic hydrogen, lose their kinetic energy dramatically so that they cannot transfer more energy to the growing surface. Thus, the diffusion length of SiH_3_ precursor cannot be enhanced. It is known that if SiH_3_ absorbed on the surface could find the energetic favorable growing sites, an atomically more ordered structure is formed. But now, SiH_3_ has not enough diffusion length to find their energetic favorable growing sites. Therefore, the ordered structure cannot be formed. In other words, the deposited film has more defects. As a result, the spin density in Fig. 5a begins to rise from 450 Pa on the contrary. However, it is worth noting that the spin densities from 600 to 1050 Pa are still lower than the ones from 150 to 300 Pa. It is the result of the weaker ion bombardment. Furthermore, as a result of its reduced diffusion length, SiH_3_ precursor tends to pile up to form aggregates. As shown in Fig. 3, aggregates began to appear at 750 Pa, and they agglomerated gradually intensively when the deposition pressure continued to increase. According to the points above, the ion bombardment is not the weaker the better for the film growth. The degree of ion bombardment should be appropriate.

### Defect Density: the Key Characteristic of nc-Si:H Photovoltaic Material

Crystallinity and defect density are both characteristics of nc-Si:H solar cell materials. The former increases with the deposition pressure. In a sense, the latter should keep declining. However, that is not the case. According to the Raman characterization, although the crystallinity increases, the variation of the grain size is very small (4.07~4.50 nm). It indicates that only the number of grains increases not the size of the grains. In these conditions, the volume of grain boundaries rises. It is known that grain boundaries are bulk defects and recombination centers. More grain boundaries will increase the defect density. When the crystallinity rises to a certain level, the negative effect of an increase in grain boundary volume on the defect density overcomes the positive effect of the rising number of grains. Therefore, the defect density does not keep decreasing as the crystallinity grows; on the contrary, it rises after the crystallinity reaches a certain level. This result suggests that nc-Si:H thin films with higher crystallinity do not necessarily have a better quality, which is confirmed by another research group. In recent years, it has been reported that the optimum nc-Si:H layer for solar cells is obtained near the phase transition boundary, i.e., the optimum is obtained just after the a-Si:H-to-nc-Si:H transition. The crystallinity of optimum nc-Si:H layers is not high [28–30]. Mukhopadhyay et al. have further demonstrated that nc-Si:H layers with high crystallinity, and thus low light-induced degradation, do not produce high-quality solar cells. The stabilized efficiency of cells deposited just after the a-Si:H-to-nc-Si:H transition is higher than the one of cells in which the i-layer has high crystallinity, although the former degrades more than the latter before stabilization [31]. Han et al. have further proven that light-induced degradation of nc-Si:H layer is introduced by the formation of metastable dangling bonds. While the light-induced structural change is a precursor process of metastable dangling bond formation [30]. The metastable dangling bond is one of the defects [32]. Therefore, the key characteristic for high-quality nc-Si:H photovoltaic materials is defect density rather than crystallinity, light stability, or other characteristics.

## Conclusions

nc-Si:H thin films were deposited by varying the pressure between 150 and 1050 Pa. The range of deposition pressure is higher than the conventional deposition in the PECVD process. It is found that crystallinity increases and the roughness of the film surfaces decreases with an increase in deposition pressure. The mean grain size *d* = 4.07~4.50 nm. Furthermore, we focused on the influence of deposition pressure not only on the macroscopic or usual properties of samples but also the defect density and minority carrier lifetime which are more important characteristics. It is found that the defect density of samples firstly decreases then increases when the deposition pressure rises. The defect density reaches its minimum (3.766 × 10^16^ cm^−3^) at 450 Pa. It is lower than that of the previous studies on the fabrication of low-defect-density nc-Si:H thin films. This work provides a convenient and effective way of depositing low-defect-density nc-Si:H by PECVD. And we have demonstrated the mechanism about the effect of deposition pressure on the ion bombardment. Moreover, it is proven that the ion bombardment is not the weaker the better for the film growth. The degree of ion bombardment should be appropriate.

## Additional file

Additional file 1: Cations passing through the anode sheath without collision. (DOCX 25 kb)

## Abbreviations

AFM: Atomic force microscope
DC: Direct current
H: Atomic hydrogen
nc-Si:H: Hydrogenated nanocrystalline silicon
PECVD: Plasma-enhanced chemical vapor deposition
SEM: Scanning electron microscopy
VHF: Very-high-frequency
