# Supplementary material for: A Convenient and Effective Method to Deposit Low-Defect-Density nc-Si:H Thin Film by PECVD
Source: Nanoscale Res Lett. 2018 Aug 10;13:234. doi: 10.1186/s11671-018-2641-z (PMC6086780; doi:10.1186/s11671-018-2641-z)
Supplement: Supplementary file 1 — Cations passing through the anode sheath without collision. (DOCX 25 kb) [file 11671_2018_2641_MOESM1_ESM.docx]

**Supplementary information**

**A convenient and effective method to** **deposit low defect density nc-Si:H thin film**

**by PECVD**

Yuwei Wang, Hong Liu, Wenzhong Shen*

Key Laboratory of Artificial Structures and Quantum Control (Ministry of Education), Department of Physics and Astronomy,

Shanghai Jiao Tong University, Shanghai 200240,

People’s Republic of China

* Corresponding author: wzshen@sjtu.edu.cn

We will confirm that cations pass through the anode sheath without collision by some calculations:

(1) The width of anode sheath

It is known that the ideal gas law is

$PV=nRT$

where P is the [pressure](https://en.wikipedia.org/wiki/Pressure) of gas, V is the volume of gas, n is the amount of substance of gas, T is the absolute temperature of gas and R is the ideal [gas constant](https://en.wikipedia.org/wiki/Ideal_gas_constant). We use N to denote the density of reaction gas.

then

$N=\frac{n}{V}=\frac{P}{\mathrm{RT}}$ (1)

The temperature of reaction gas before discharge approximately equals room temperature. The deposition pressure is from 150 Pa to 1050 Pa, thus taking P=150 Pa, we obtain: the minimum of N, N_min_=$3.6204\times{10}^{16}\mathrm{cm}^{-3}$.

We define μ as the ionization rate, n_e_ as the electron density, then

$n_{e}=N\cdot\mu$ (2)

In the plasma without DC bias, the width of sheath is equal to the debye length which is [[1](#_ENREF_1)]

$\lambda_{D}=\sqrt{\frac{\varepsilon_{0}kT_{e}}{n_{e}e^{2}}}=6.9\sqrt{\frac{T_{e}}{n_{e}}}$ (3)

where $\varepsilon_{0}$ is the vaccum dielectric constant, k is the Boltzmann constant, T_e_ is the electron temperature, n_e_ is the electron density, e is the quantity of electric charge, T_e_: K, n_e:_ cm^-3^, $\lambda_{D}$: cm.

As the plasma generated by RF of 13.56 MHz belongs to the weak ionized one, $\mu$ is between 0.001% - 1% [[1](#_ENREF_1)]. The range of the electron temperature is $5\times{10}^{3}-2\times{10}^{4} K$ because the discharge in our reactor is one type of glow discharge [[2](#_ENREF_2)].

According to formula (3), we will get the maximum of $\lambda_{D}$ ($\lambda_{\mathrm{Dmax}}$), taking the maximum of T_e_ and the minimum of n_e_. Thus, T_e_=$2\times{10}^{4} K$ and $n_{e}=N_{\min}\cdot\mu$ would be used. We take two minimal value of $\mu$ as follows:

When$\mu$=0.001%, $\lambda_{\mathrm{Dmax}}=16.2176 \mu m$;

$\mu$=0.01%, $\lambda_{\mathrm{Dmax}}=5.1284 \mu m$;

that is, the maximum of anode sheath width is between $5.1284-16.2176 \mu m$.

(2) The minimal mean free path of cation

It is known that the mean free path of gas molecule is

$l=\frac{\mathrm{kT}}{\sqrt{2}\pi d^{2}p}$ (4)

The mean free path of cation and the one of gas molecule, their order of magnitude are equal. We could thus use formula (4) to calculate the order of magnitude of the mean free path of cations which pass through the anode sheath (l_c_). Then,

$l_{c}=\frac{\mathrm{kT}}{\sqrt{2}\pi d^{2}p}$ (5)

where d is the diameter of cations and p is the deposition pressure. Although the one of electron is dramatically high, the temperature of ion is little higher than room temperature. We take T=300 K(room temperature) to obtain the minimum of l_c_; The main cations are $\mathrm{Si}H_{x}^{+}, H^{+}$ in the plasma [[3](#_ENREF_3)], thus we should use the diameter of $\mathrm{Si}H_{x}^{+}$ which equals to one-fiftieth of $\mathrm{Si}_{50}H_{n}$ (n is arbitrary number) in order to get the minimum of l_c_; that is, taking d=$\frac{1}{50}$ nm (the diameter of $\mathrm{Si}_{50}H_{n}$ is about 1nm [[4](#_ENREF_4)]), p=1050 Pa (the maximum of deposition pressure), T=300 K, we obtain:

the minimum of l_c_, l_cmin_=0.2220 cm, i.e. the order of magnitude of the minimal mean free path of cation passing through the anode sheath is millimeter.

(3) Comparing $\lambda_{\mathrm{Dmax}}$ with $l_{\mathrm{cmin}}$, it is obvious that the minimal mean free path of cation is far bigger than the maximum of the anode sheath’s thickness. Therefore, it is confirmed that cations pass through the anode sheath without collision.

**References**

1. Lieberman MA, Lichtenberg AJ. Principles of plasma discharges and materials processing. Second ed: John Wiley & Sons; 2005.

2. Eliasson B, Kogelschatz U. Nonequilibrium Volume Plasma Chemical Processing. Ieee transactions of plasma science. 1991;19:1063-77.

3. Matsuda A, Takai M, Nishimoto T, Kondo M. Control of plasma chemistry for preparing highly stabilized amorphous silicon at high growth rate. Solar Energy Materials and Solar Cells. 2003;78(1-4):3-26.

4. Gallagher A, Bano G, Rozsa K. Particles in silicon deposition discharges. Solar Energy Materials and Solar Cells. 2003;78(1-4):27-40.
